# Tannin an Antidote of Strychnia.—Kursak

**Published:** 1861-06

**Authors:** 


Tannin an Antidote of Strychnia.—Dr. Kursak concludes, from numerous experiments on dogs and rabbits, that the prompt exhibition of tannin is the safest and most efficient remedy for poisoning by strychnia. The quantity of the antidote should be proportionate to the amount of the poison which has been taken, and even somewhat exceed the latter, inasmuch as the contents of the stomach are liable to absorb a portion of the neutralizing agent. For every grain of strychnia, about two drachms of gall-nuts, or more in case of vomiting, should be prescribed. Green tea also appears to possess a certain degree of efficacy, but only when the quantity of poison is very small. Oak-bark, acorns, etc., are convenient and active substitutes for pure tannin. But vegetable acids should be prohibited during the treatment, as they promote the dissolution of the precipitates thrown down under the influence of the tannic acid—the same exception applies equally to fermented liquids; and muscular efforts which induce convulsive action, in persons poisoned with strychnia, should likewise be carefully avoided.—*L'Union Médicale.*

